# Effect of Hemiepiphysiodesis on the Growth Plate: The Histopathological Changes and Mechanism Exploration of Recurrence in Mini Pig Model

**DOI:** 10.1155/2018/6348171

**Published:** 2018-12-30

**Authors:** Jing Ding, Jin He, Zhi-qiang Zhang, Zhen-kai Wu, Fang-chun Jin

**Affiliations:** ^1^Department of Pediatric Orthopaedics, Xinhua Hospital, School of Medicine, Shanghai Jiao Tong University, No. 1665, Kongjiang Road, Shanghai 200092, China; ^2^Division of Orthopaedic Surgery, Children's Mercy Kansas City, 2401 Gillham Road, Kansas City, MO 64108, USA

## Abstract

**Purpose:**

Hemiepiphysiodesis has been widely used to correct angular deformity of long bone in immature patients. However, there is a limited knowledge about the biomechanical effect of this technique on the histopathological changes of the growth plate and the mechanism of recurrence of malformation after implant removal. We aimed to evaluate the biomechanical effect of hemiepiphysiodesis on the histopathological changes of the growth plate and the mechanism of recurrence of malformation after implant removal in Bama miniature pigs, and to explore the role of asymmetric stress during this procedure.

**Methods:**

Eight 3-month-old male Bama miniature pigs sustained surgeries on the bilateral medial hind leg proximal tibia as the intervention group (n=16), and four pigs sustained bilateral sham surgeries as the control (n=8). In the 18^th^ week after surgeries, hardware was removed in the unilateral leg of each animal in the intervention group. In the 24th week of the study, all animals were euthanized. A total of 24 samples were obtained and stained with H&E, TUNEL, and immunohistochemistry. Sixteen samples in the intervention group were divided into two subgroups. The tibias without an implant were included in the implant removal group (IR group), while the tibias with an implant were included in the implant persist group (IP group). The proximal tibia specimens were divided into 3 equidistant parts from medial to lateral, named as area A, area B, and area C, respectively. The change of thickness of growth plates, chondral apoptosis index, and the expression of Caspase-3, Caspase-9, CHOP, and P65 were compared.

**Results:**

H&E staining showed the thickness of growth plate to be varied in different areas. In the IP group, the thickness of growth plate in areas A and B was statistically significantly thinner than that in area C (p<0.05). In the IR group, the thickness of growth plate in areas A and B was statistically significantly thicker than that in area C (p<0.05). TUNEL staining showed that the apoptosis rate increased significantly after hemiepiphysiodesis and declined after implant removal (p<0.05). Immunohistochemical staining suggested that the expression of Caspase-3, Caspase-9, P65, and CHOP protein was upregulated in the experimental group and downregulated after implant removal.

**Conclusion:**

The thickness parameter of the growth plate changes with asymmetric pressure. When the pressure is relieved, the recurrence of malformation is related to the thickening of the growth plate.

## 1. Introduction

During childhood, long bones consist of osseous tissue and the cartilaginous growth plate responsible for their axial growth [1]. Any factor that interferes with the osteogenetic process of the local growth plate, such as trauma, bone dysplasia, endocrine and metabolic diseases, inflammation, or Blount's disease, may cause angular deformity and imbalance in limb growth. Theoretically, temporary hemiepiphysiodesis has the ability to temporarily arrest the growth of target physis, with progressively normal growth occurring out to the far edge of the growth plate and finally correcting the angular deformity [2–7].

The mechanism by which hemiepiphysiodesis regulates endochondral ossification is unclear. Stress acting on the growth plate has been confirmed to play an important role in regulating the process of endochondral ossification [8, 9]. It is known that increased compression slows growth, and decreased compression or distraction accelerates growth, according to the Hueter-Volkmann principle. Bylski-Austrow et al. reported that the growth plate exerted stresses (about 1 MPa) in the process of hemiepiphysiodesis [10]. Stoke et al. [11] reported an apparently linear negative relationship between stress and percentage growth modulation in animal studies, but no clinical research confirms this finding [12]. Congdon et al. [13] illustrated that chondral modeling may be subject to a threshold effect, with articular and physeal cartilage reaching a response threshold at different force magnitudes. Histopathological images showed the thickness of the proliferative layer and the hypertrophy layer of the cartilage to be significantly thinner than that of the control group secondary to continuous compressive stress [8, 14, 15]. Different from the above research model, the stress acting on the cartilage in the hemiepiphysiodesis process is not a uniform but an asymmetric distribution [2]. Another issue is the recurrence of malformations that occurs after implant removal according to previous studies [2, 5, 6, 16, 17]. Zuege and other researchers believe that the recurrence of these malformations is spontaneous and unavoidable in animal experiments [3, 18]. The mechanism of malformation recurrence is still unclear.

We hypothesize that the stress acting on the growth plate is asymmetrical when the process of hemiepiphysiodesis is asymmetrical. The purpose of this study is to evaluate the biomechanical effect of hemiepiphysiodesis on the histopathological changes of the growth plate and the mechanism of recurrence of malformation after implant removal in Bama miniature pigs and to explore the role of asymmetric stress during this procedure.

## 2. Materials and Methods

### 2.1. Animals and Grouping

**Figure 1:**
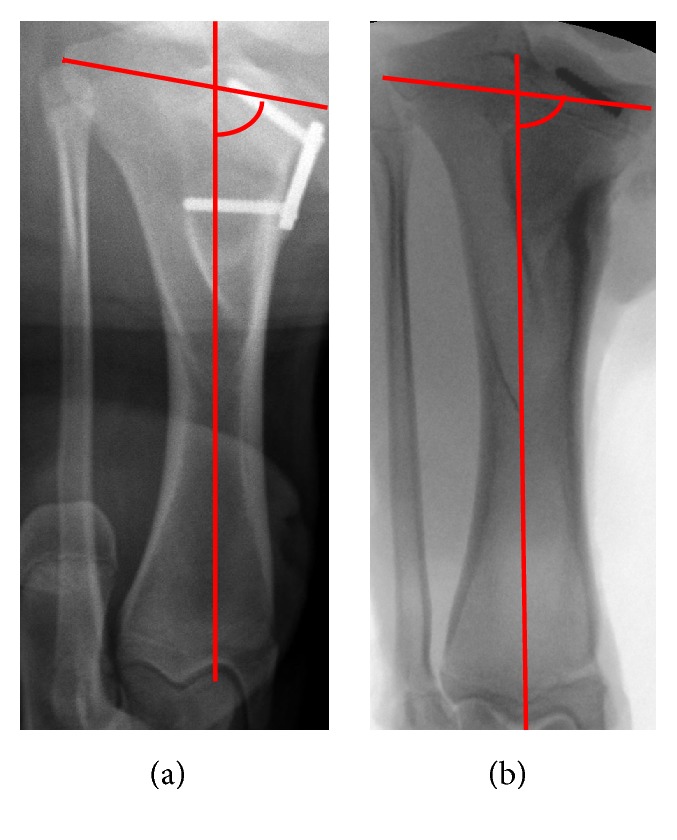
The tibial image of animal model ((a) 18 weeks after operation; (b) 6 weeks after removal of the internal fixation. The red arc is the media proximal tibial angular (MPTA) angle).

We got approval from the Institutional Review Board/Ethics Committee, Xinhua Hospital. A total of twelve 3-month-old male Bama miniature pigs were used for this study, eight of them (16 tibias) as the intervention group and four (8 tibias) as the control group. The intervention group sustained surgeries with a tension band plate (Shanghai Puwei Medical Instrument Co., Ltd., Shanghai, China) at the bilateral medial hind leg proximal tibia. The control group sustained sham surgeries at the same position. The surgical technique is the same as the procedure described in our previous study [19]. By the use of fluoroscope, the implants were placed carefully to keep the periosteum and growth plate intact. The implant in the unilateral tibia (assigned at random) of each animal in the intervention group was removed 18 weeks later postoperatively. In the 24th week of the study, all animals were euthanized. A total of 24 tibia samples were extracted. Sixteen samples from the intervention group were divided into two subgroups. The tibias without implant samples were included in the implant removal group (IR group), while the tibias with implant samples were included in the implant persist group (IP group). We found that, for 6 weeks after the internal fixation removal, the media proximal tibial angular (MPTA) angle was significantly larger than that before the removal (Figure 1).

For all samples, 6 sections were obtained along the growing axis and stained with H&E, TUNEL, and immunohistochemistry. The change in thickness of physeal plates, chondral apoptosis index, and the expression of Caspase-3, Caspase-9, CHOP, and P65 (Dako Diagnostics Inc., Mississauga, ON, Canada) were compared.

### 2.2. Analysis

**Figure 2:**
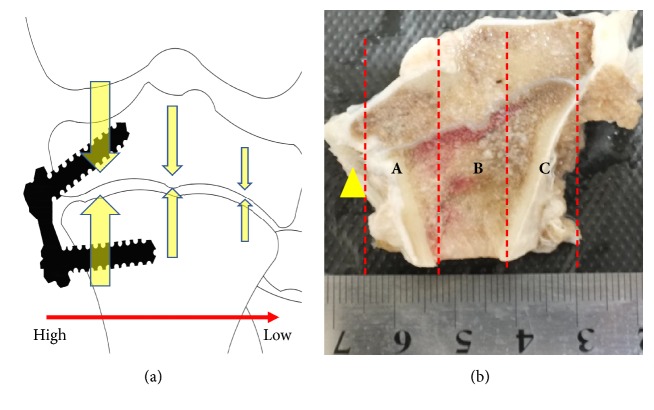
Sample of proximal tibia ((a) sketch map showed that the stress acting on the cartilage in the hemiepiphysiodesis process was an asymmetric distribution; (b) the yellow arrow points to the surgical site. The proximal tibia specimen was divided into 3 parts from the inside to the outside: A, B, and C).

We used the NIKON BR3.5 software to calculate and analyze the morphology of the different layers (resting, proliferative, and hypertrophic), the apoptotic index of the plate cells at different time points, and the positive staining rate of cells. We divided the proximal tibia specimen into 3 equidistant parts from medial to lateral, named area A, area B, and area C, respectively (Figure 2). Each of the 3 areas was selected for 3 sections (7-*μ*m thickness) for measurement. Using HE stained sections, 6 points were consecutively measured at a field of view of 100X (1000*μ*m per point). In Tunel and immunohistochemical staining, the percentage of positive cells in total cells was measured in 6 fields at 200 X magnification. Measurement of 3 slices for each measurement index was repeated to get the average value. The entire measurement process was performed by 2 orthopedists trained in histopathological measurements. Spearman correlation coefficient was used to evaluate intra- and interclass correlation coefficient (ICC). The paired t-test and the 95% confidence interval were used to compare the measurements between the different groups. All analyses were performed with the Stata software (version 12.0, Stata, USA), and p < 0.05 was considered statistically significant.

## 3. Results

In the IP group, HE staining suggested that area A was the thinnest part of the growth plate, which had an irregular shape; the proliferative layer disappeared; the cell arrangement was disordered; and the cell morphology was anomalous. Area B was thicker than area A. The shape of growth plate was also irregular, the columnar structure of proliferative layer existed, and the arrangement of cells was slightly disordered. Area C was the thickest part of the growth plate, which had a basically normal shape. The structure of the proliferative layer was arranged regularly.

**Figure 3:**
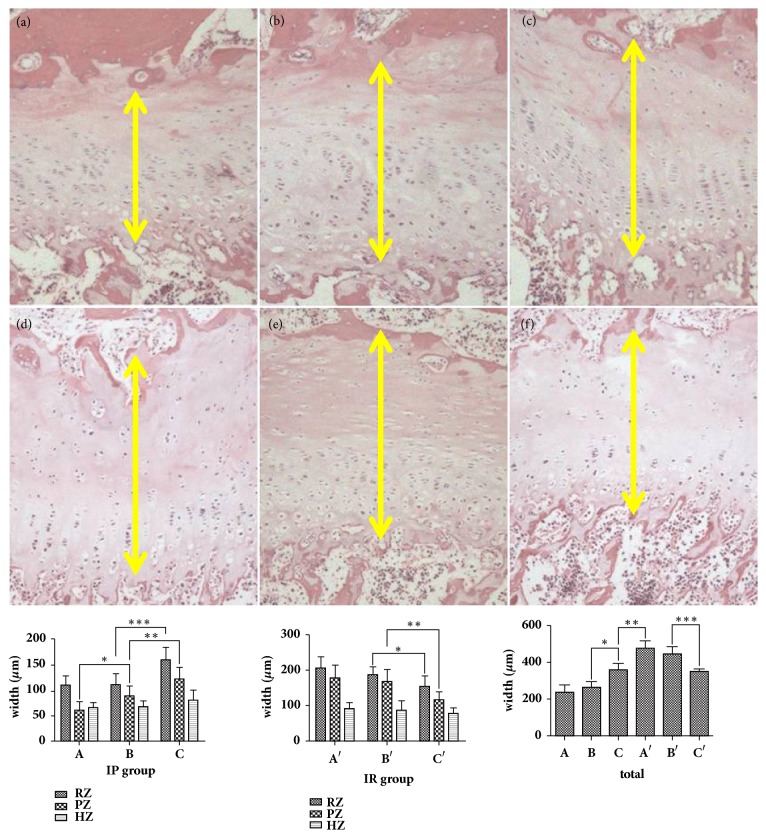
The thickness of growth plate in different area (representative H&E staining showing growth plate thickness (a) A area in the IP group; (b) B area in the IP group; (c) C area in the IP group; (d) A area in the IR group; (e) B area in the IR group; (f) C area in the IR group; yellow double arrow: the thickness of the growth plate; IP group: *∗*=0.0089; *∗∗*=0.008; *∗∗∗*= 0.0011; IR group: *∗*=0.0023; *∗∗*=0.0019; total width of growth plate: *∗*≤0.001; *∗∗*≤0.001; *∗∗∗*≤0.001; RZ: resting zone, PZ: proliferative zone, HZ: hypertrophic zone, 100 X magnification).

In the IR group, the thickness and the shape of growth plate in A were similar to B, and the arrangement of cells was disordered. In area C, the growth plate was thinner than that in A and B, and the morphology was normal. The columnar structure was similar to that in area C of the IP group (Table 1) (Figure 3).

Our measurements revealed that in the IP group the thickness of the growth plate from area A to area C was increased gradually and that there was a statistically significant difference between areas B and C (p≤0.001). Measurement of each layer showed a statistically significant difference in the proliferative layer between area A and area B (p=0.0089), and between area B and area C (p=0.0080). In the resting layer, we observed no statistical difference between area A and area B, but a significant difference between area B and area C (p=0.0011). We found no statistically significant difference in the thickness of the different hypertrophy layers.

**Figure 4:**
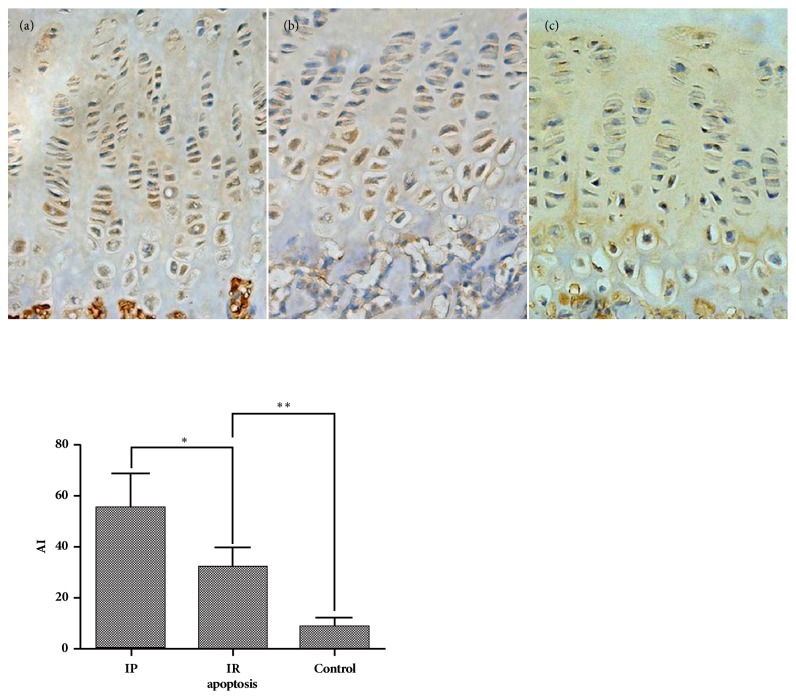
The apoptosis of proximal tibial growth plate ((a) IP group, (b) IR group, and (c) control group; *∗*: p≤0.001; *∗∗*: p≤0.001, 200 X magnification).

**Figure 5:**
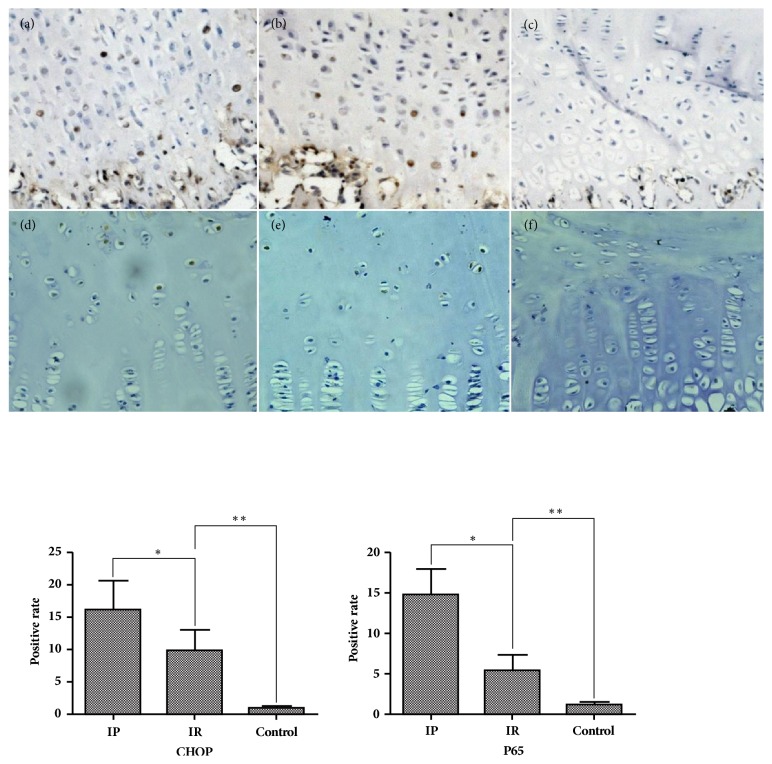
The expression of CHOP and P65 in immunohistochemical staining ((a), (b), (c) CHOP staining; (d), (e), (f) P65 staining; (a), (d): IP group; (b), (e): IR group; (c), (f): control group; *∗*: p=≤0.001; *∗∗*:p≤0.001, 200 X magnification).

**Figure 6:**
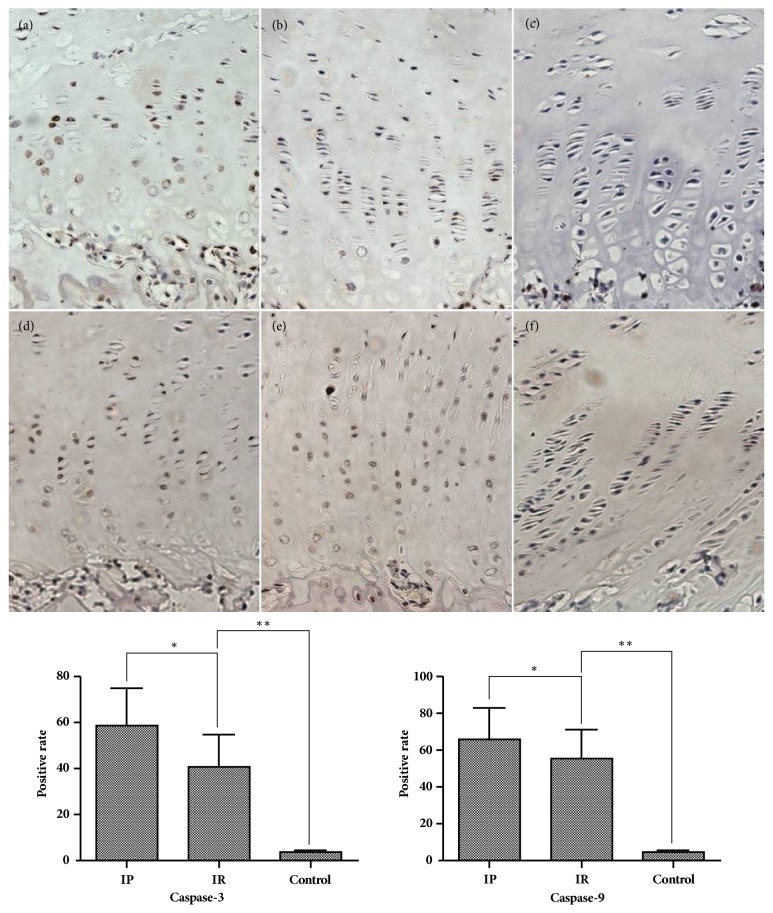
The expression of Caspase-3 and Caspase-9 in immunohistochemical staining ((a), (b), (c) Caspase-3 staining; (d), (e), (f): Caspase-9 staining; (a), (d): IP group; (b), (e): IR group; (c), (f): control group; Caspase-3 *∗* p≤0.001; *∗∗*p≤0.001; Caspase-9 *∗*p=0.0357; *∗∗*p=≤0.001, 200 X magnification).

In the IR group, the thickness of growth plate from area A to area C showed a decreasing tendency. The thickness of area B was significantly higher than that of area C (p≤0.001). The thickness of the resting and proliferative layers in area B was significantly higher than the resting layer (p=0.0023) and proliferative layer (p=0.0019) in area C; however, the thickness of the hypertrophy layer was almost unchanged. We also found that the thickness of area A in the IR group was significantly higher than that of area C in the IP group (p≤0.001), but there was no statistical difference in area C between the IR group and the IP group (Figure 3). The apoptotic index in the IP group was significantly higher than that in the IR group (P≤0.001), which was significantly higher than the control group (p≤0.001) (Figure 4). The percentage of positive cells for CHOP, P65, and Caspase-3 and Caspase-9 in the IP group was significantly higher than that in the IR group (P≤0.001), which was significantly higher than the control group (p≤0.001) (Figures 5 and 6). The Spearman intra- and interclass correlation coefficients were 0.85 and 0.88, respectively.

## 4. Discussion

In our previous study [19], we compared the correction effect of different type of implant during hemiepiphysiodesis. In this study, we built a similar animal model and found that the gradual change of the thickness of the growth plate associated with hemiepiphysiodesis was highly related to the characteristics of asymmetrical pressure distribution we hypothesized above [2]. After pressure release, the phenomenon of catch-up growth is related to the thickening of the growth plate and is inevitable. We also found a significantly increased apoptosis rate after hemiepiphysiodesis, which declined after implant removal (p<0.05). Expression of Caspase-3, Caspase-9, P65, and CHOP protein was upregulated in the implant persist group and downregulated after implant removal. These findings verified our hypothesis and revealed that the asymmetrical pressure acting on the growth plate plays an important role in adjusting ossification rates in different parts of the plate.

It has been generally accepted that the elongation of long bones is the result of the combined effects of proliferation, hypertrophy, apoptosis, extracellular matrix synthesis, degradation, and calcification of chondrocytes. The endochondral growth rate is closely related to the proliferation rate of chondrocytes, the height of hypertrophy, and the synthesis rate of matrix in the hypertrophic layer [8, 15, 20, 21]. For longitudinal (linear) growth, Stokes et al. [22] proposed the formula, growth = new cells/day x final height, which represents this presumed relationship between growth and the measured activity of cell production in the proliferative zone and cell enlargement in the hypertrophic zone. In another report, Stokes et al. demonstrated a linear relationship between mechanical compression and epiphysis growth rate. When the pressure increased, the growth rate of the chondrocytes decreased, and vice versa [11]. Based on the above formulas, it can be concluded that the growth produced by sustained mechanical load is influenced by both the regulation of hypertrophy and alterations in the rate of chondrocytes' proliferation and might eventually lead to the growth plate being compressed or even stopped. Previous research has also confirmed this conclusion [8, 14, 15]. We speculated that the degree of structural changes should differ in different parts of the growth plate during hemiepiphysiodesis. Based on this hypothesis, we tested the three areas of A, B, and C from near and far distances for individual observations of the growth plate. The results showed gradual enlargement of the thickness of the growth plate from area A to area C, contrary to the characteristics of pressure distribution. The thickness of the proliferating layer presented a similar phenomenon, while no significant tendency was found in the hypertrophic and resting layer, suggesting that the proliferative layer may be more susceptible to the stress.

When the catch-up growth is limited to one side of the growth plate, as occurs after hemiepiphysiodesis, there may be a loss of correction. In previous animal studies, when the internal fixation was removed, the growth plate on the arrested side produced accelerated growth [3, 18]. Histological studies suggested that the growth plate was significantly thicker than that of the control group when the compressive stress was relieved. The COL (collagen) II and COL X expression demonstrated wider expression zones in the experimental group than in the control. The upregulation of OPN (osteopontin) and ALP (alkaline phosphatase) in the growth plates after load release was associated with accelerated ossification concurrent with the catch-up growth. This phenomenon of “catch-up growth” is the result of self-adjustment of the epiphyseal plate and may be related to the recurrence of abnormalities after the removal of the tension band plate [23, 24]. In this study, we found a gradual thinning of the growth plate from area A to area C, in which the thickness of area C was similar to the IP group, while area A was markedly thickened compared to the IP group. The measurement of the deferential layer of the growth plate showed the intensive change of the proliferative layer, which suggested that, after pressure release, the phenomenon of catch-up growth is related to the thickening of the growth plate, and the thickening of the proliferative layer plays the most important role.

Studies have confirmed that apoptosis of cells in the hypertrophy layer is closely related to endochondral bone formation [25, 26]. The apoptotic cells and the apoptotic proteins Caspase-3 and Caspase-9 after hemiepiphysiodesis were recorded by our research. The results showed that the apoptotic index of the cells in the IP group was significantly increased, and the positive rate of apoptotic proteins increased, while in the IR group the positive rate of apoptotic protein was significantly decreased, but was still significantly higher than the control group. Physiologically, the apoptosis of the epiphyses cells mainly occurs at the interface between the hypertrophic layer and the bone, and the apoptosis of mature hypertrophic cell promotes the cartilage calcification and ossification process. However, in this study, when the proximal tibial growth plate sustained surgeries with an implant, apoptosis occurred in the whole hypertrophic layer and in the local proliferative layer adjacent to the hypertrophic layer. Nevertheless, following implant removal, the expression of apoptotic index and apoptotic protein began to decline. We suspect that, as a result of hemiepiphysiodesis, premature apoptosis occurred in some proliferative cells that skipped the process of hypertrophy, subsequently interfering with cartilage calcification and endogenous ossification.

Some studies have found that although the expression of matrix-related proteins changes under pressure, the mRNA expression does not change significantly, suggesting that still unknown mechanisms are regulating endochondral ossification [27, 28]. Recent studies have confirmed that endoplasmic reticulum stress (ERs) plays an important role in proliferation, differentiation, and regulation of endogenetic ossification in chondral chondrocytes [28, 29]. Abnormal secretion and folding of matrix protein precursors caused by any reason can cause the accumulation of proteins in the endoplasmic reticulum and damage the normal physiological functions of the endoplasmic reticulum, producing ERs. When continuously activated, ERs will activate the signal of endoplasmic reticulum apoptosis and upregulate the expression of CHOP, leading to the apoptosis of the cells [30]. On the other hand, the endoplasmic reticulum senses ERs through three transmembrane endoplasmic reticulum proteins: PERK (protein kinase RNA-like ER kinase), ATF6 (activation transcription factor 6), and IRE1 (inositol requiring enzyme-1). These transcription factors increase the ability of the endoplasmic reticulum to fold other proteins by upregulating protein chaperones and endoplasmic reticulum-related degradation by-pass components to help improve ERs, but at the same time interfere with cartilage hypertrophy and endochondral ossification [29, 31]. Both PERK and IRE1 can activate the IKK-NF*κ*B signaling pathway [32]. Activated NF-*κ*B translocates into the nucleus and regulates expression of genes involved in cell growth, adhesion, and death. In recent years, many studies have confirmed that the NF-*κ*B p65 pathway has the effect of improving ERs and is closely related to the ossification of cartilage and the growth of long bones. It also acts through BMP-2 [33, 34]. In this study, the expression of CHOP and P65 in the IP group was significantly increased. We speculate that stress affected the synthesis and secretion of extracellular matrix proteins. The secretion of matrix proteins in the growth plate decreases, and excessive precursor proteins accumulate in the cells, subsequently causing ERs. Sustained pressure eventually activates the apoptotic pathway, leading to premature apoptosis of chondral chondrocytes, while the endoplasmic reticulum improves ERs through the UPR pathway and activates the NF-*κ*B P65 pathway that acts synergistically to improve stress in this process. Six weeks after the release of stress, the apoptosis index and the expression of CHOP protein in the Tunel cells were gradually decreased but still significantly higher than those in the control group. The positive rate of P65 also showed a similar trend. We speculate that, after the releasing load, the synthesis of proteins in chondral chondrocytes recovered, and the function of ERs was restored, but this improvement was a gradual process. Activation of the NF-*κ*B P65 pathway promotes cartilage ossification and long bone growth at the same time as synergistic relief of stress, which may be one of the reasons for the “catch-up growth.”

In this study, we used the 3-month-old Bama miniature pig as a test animal. Compared to the more commonly used animal models such as rats and rabbits, the long bone plate structure of the miniature pig is closer to the human growth plate [35]. Our findings implicate that, by the use of stress controlling, we might treat different kinds of angular deformity more efficiently in clinic. A prophylactic overcorrection of angular deformity is meaningful to deal with the inevitable catch-up growth [2–6]. The apoptosis and ERs pathway might be important in this procedure. Novel biological targets for the therapy of angular deformity will be considered in the future.

The limitation of this study is that it provides only an initial observation of the changes in the morphological structure, apoptotic index, and immunohistochemistry-related protein changes under the hemiepiphysiodesis. Research is still needed to investigate the relationship between morphological changes of growth plate and endogenous ossification rate and the participation of apoptosis and the ERs pathway in the regulation of endogenetic ossification.

## 5. Conclusion

The thickness parameter of the growth plate changes with the asymmetric pressure: the greater the pressure, the thinner the growth plate, and vice versa. When the pressure is relieved, the recurrence of malformation is related to the growth plate thickening. The stress mainly influences the proliferative layer.

## Figures and Tables

**Table 1:** The thickness of growth plate in two groups (Unit: *μ*m).

**Groups**	**layer**		**A**	**B**	**C**
**IP group**	R		109.42±18.72	110.23±22.53	159.29±25.48
		VS B*∗*	P>0.05	/	p=0.0011
	P		61.61±15.52	88.50±19.65	121.71±23.18
		VS B*∗*	p=0.0089	/	p=0.0080
	H		65.58±10.73	67.90±11.47	80.98±19.37
		VS B*∗*	P>0.05	/	P>0.05

**IR group**	R		206.19±32.89	187.64±21.58	155.24±28.68
		VS B*∗*	P>0.05	/	P=0.023
	P		178.04±37.57	169.27±32.17	115.65±23.49
		VS B*∗*	P>0.05	/	P=0.0019
	H		90.26±18.77	87.70±25.34	78.93±13.22
		VS B*∗*	P>0.05	/	P>0.05

R: resting layer; P: proliferative layer; H: hypertrophy layers; A: A area; B: B area; C: C area

*∗*All P values were calculated by comparing with B.

## Data Availability

The data used to support the findings of this study are available from the corresponding author upon request.
